# Transcription Factor *MaMsn2* Regulates Conidiation Pattern Shift under the Control of MaH1 through Homeobox Domain in *Metarhizium acridum*

**DOI:** 10.3390/jof7100840

**Published:** 2021-10-07

**Authors:** Dongxu Song, Yueqing Cao, Yuxian Xia

**Affiliations:** 1 School of Life Sciences, Chongqing University, Chongqing 401331, China; songdongxu@yeah.net; 2 Chongqing Engineering Research Center for Fungal Insecticides, Chongqing 401331, China; 3 Key Laboratory of Gene Function and Regulation Technologies under Chongqing Municipal Education Commission, Chongqing 400044, China

**Keywords:** *Msn2*, normal conidiation, microcycle conidiation, dimorphism, entomopathogenic fungi

## Abstract

The growth pattern of filamentous fungi can switch between hyphal radial polar growth and non-polar yeast-like cell growth depending on the environmental conditions. Asexual conidiation after radial polar growth is called normal conidiation (NC), while yeast-like cell growth is called microcycle conidiation (MC). Previous research found that the disruption of *MaH1* in *Metarhizium acridum* led to a conidiation shift from NC to MC. However, the regulation mechanism is not clear. Here, we found *MaMsn2*, an *Msn2* homologous gene in *M. acridum*, was greatly downregulated when *MaH1* was disrupted (Δ*MaH1*). Loss of *MaMsn2* also caused a conidiation shift from NC to MC on a nutrient-rich medium. Yeast one-hybrid (Y1H) and electrophoretic mobility shift assay (EMSA) showed that MaH1 could bind to the promoter region of the *MaMsn2* gene. Disrupting the interaction between MaH1 and the promoter region of *MaMsn2* significantly downregulated the transcription level of *MaMsn2*, and the overexpression of *MaMsn2* in Δ*MaH1* could restore NC from MC of Δ*MaH1*. Our findings demonstrated that *MaMsn2* played a role in maintaining the NC pattern directly under the control of MaH1, which revealed the molecular mechanisms that regulated the conidiation pattern shift in filamentous fungi for the first time.

## 1. Introduction

Filamentous fungi have dimorphism. The cell can switch between radial polar growth and non-polar yeast-like cell growth depending on the external environmental conditions, including temperature, nutrients, carbon dioxide concentration, pH and other conditions [1,2,3]. 

Conidia are the life initiation and termination units of filamentous fungi. The stress resistance of conidia is generally higher than that of vegetative cells. Some fungal spores are among the most stress-resistant eukaryotic cells described so far [4]. There are two patterns of asexual conidiation in filamentous fungi: normal conidiation (NC) and microcycle conidiation (MC) [5]. NC occurs after radial polar growth, while non-polar yeast-like cell growth is called MC. In MC, the fungi develop secondary conidia on conidiophores produced from germ tubes or directly from conidial cells. MC usually occurs under unfavorable environmental conditions, such as nutrient deficiency. MC was originally reported in *Aspergillus niger* and has been found in more than 100 fungi, including entomopathogenic fungi [5,6,7]. 

Compared with NC, MC has more advantages in mass production and field application, for example, increased conidial yield, improved conidial stress tolerance and more uniform conidia in size [8]. The NC regulation pathway has been thoroughly studied in *Aspergillus*. Transcription factors *BrlA*, *AbaA* and *WetA* constitute the core regulatory pathway of NC in *Aspergillus nidulans* [9]. Disrupting any of these three genes can block conidiation [10,11,12]. In addition, upstream regulatory genes, such as *FluG* and *FlbA-E*, also regulate the core regulatory pathway of conidiation [13]. Compared with NC, only a few genes involved in MC have been identified. In *Fusarium graminearum,* the absence of *WetA* causes longer conidia and the fungi directly produce the second conidia from conidia without mycelium formation, showing MC characteristics [14]. In *M. acridum*, the downregulation of the *mmc* gene leads to a conidiation pattern shift from MC to NC with significantly decreased growth and conidia yield [15]. Disruptions of *PepdA* and *MaCreA* also lead to a shift in conidiation pattern from MC to NC [16,17].

Homeobox genes, which can bind to DNA by the homeobox domain or homeodomain containing a helix-turn-helix motif, are an important class of transcription factors in eukaryotes [18]. Homeobox genes have been reported to be involved in conidiation processes among different fungi. For instance, *PahI* plays a negative role in microconidiogenesis in *Podospora anserina* [19]. The *PahI* homologous gene *Bchox8* is also involved in mycelial development and conidiation in *Botrytis cinerea* [20]. *Mohox4* and *Mohox6* can affect conidial size and hyphal development in *Magnaporthe oryzae* [21]. A previous study showed that the deletion of the homeobox gene *MaH1* resulted in a conidiation shift from NC to MC in *M. acridum* when cultured on a nutrient-rich medium 1/4 SDAY [22]. However, the mechanisms that regulate the shift have not been reported. In this study, we found that the multicopy suppressor of *snf 2* (also named *Msn2*) was downregulated in *MaH1-*deficient *M. acridum*, suggesting that *MaH1* might regulate *Msn2*.

Msn2, a C_2_H_2_ transcription factor, is regulated by PKA phosphorylation and located in the cytoplasm under normal circumstances. After being stimulated by environmental stresses, such as severe temperature, osmotic and oxidative stresses, Msn2 is rapidly dephosphorylated by PP1 protein phosphatase and translocated into the nucleus [23,24]. Msn2 is also regulated by genes involved in the MAPK pathway [25], Ras–cAMP–PKA pathway [26], Snf1 protein kinase pathway [27], TOR pathway [28] and GSK-3 homologs activity pathway [29]. In the nucleus, *Msn2* regulates the transcription of a large number of stress response-related genes by binding to the stress response element in the promoter region [30]. The roles of *Msn2* vary in different fungi. *Msn2* in *B. bassiana* (*BbMsn2*) and *M. robertsii (MrMsn2)* contribute to conidiation, multiple stress responses and virulence [31]. *Bbmsn2* is a pH-dependent negative regulator, which regulates secondary metabolism and produces a red pigment called oosporein [32]. *Msn2* in *Magnaporthe oryzae* (*MoMsn2*) affects aerial hyphal growth, conidiation and virulence. *MoMsn2* is targeted by mitogen-activated protein kinase *MoOsm1* and interacts with downstream gene *MoCos1* in the osmotic regulation pathway [33]. *Msn2* in *Verticillium dahliae* (*VdMsn2*) controls mycelial growth, microsclerotia formation and virulence [34].

In this study, we focused on the regulation mechanism of MaH1 and *Msn2* in the conidiation pattern shift in *M. acridum* and found that *MaMsn2* played a role in maintaining the NC pattern directly under the control of MaH1.

## 2. Materials and Methods

### 2.1. Strains and Culture Conditions

Wild type *M. acridum* CQMa102 (WT) was deposited in the China General Microbiological Culture Collection Center (CGMCC; No. 0877; GCF_000187405.1). The WT, knockout and complement strains were cultured on nutrient-rich medium 1/4 SDAY (10 g glucose, 2.5 g peptone, 5 g yeast extract, 18 g agar and 1000 mL water) for normal growth and conidiation for 15 days at 28 °C in the dark. Microcycle conidiation was observed on nutrient-limited medium SYA (30 g sucrose, 5 g yeast extract, 3 g NaNO_3_, 5 g MgSO_4_, 5 g KCl, 1 g KH_2_PO_4_, 0.01 g FeSO_4_, 0.01 g MnSO_4_, 18 g agar and 1000 mL water). *Agrobacterium tumefaciens* AGL-1 and *Escherichia coli* (*E. coli*) DH5α were purchased from Bioground (Beijing, China) and cultured on Luria-Bertani medium (LB).

### 2.2. Bioinformatic Analysis of Genes

The gene and protein sequences were derived from NCBI at https://www.ncbi.nlm.nih.gov/ (accessed on 12 November 2019). The isoelectric point (pI) and molecular weight were predicted at https://web.expasy.org/protparam/ (accessed on 12 November 2019). Conserved domains of target genes were predicted using NCBI Conserved Domain Search at https://www.ncbi.nlm.nih.gov/Structure/cdd/wrpsb.cgi (accessed on 12 November 2019).

### 2.3. Construction of the Mutant Strains 

The target gene knockout strains were generated by homologous recombination. About 1 kb fragments of the 5’ and 3’ flanking regions of the target gene were amplified from WT genomic DNA via PCR, and the two fragments were inserted into PK_2_-PB vector with a *bar* gene as selection marker to obtain PK2-5’-bar-3’ knockout vector. The vector was introduced into wild type strain by *Agrobacterium*-mediated transformation [35], and the target gene was replaced with the *bar* gene. For the complement strains, the 5’ promoter region and the coding region of the target gene were amplified together from WT genome via PCR and inserted into the PK_2_-Sur vector to obtain PK_2_-Sur-CP [36]. The complementary vector was transformed into the knockout strains by the *Agrobacterium*-mediated method. The target gene was inserted into the genome of the knockout strains to obtain complement strains. Both knockout and complement strains were verified by Southern blot using the DIG High Prime DNA Labeling and Detection Starter Kit I (Cat. No. 11585614910, Roche, Germany). For the *MaMsn2*:*egfp* strain and Δ*MaH1*:*MaMsn2*^OE^ (*MaMsn2* overexpressed with a constitutive glyceraldehyde 3-phosphate dehydrogenase promoter (*gpd*) in Δ*MaH1*), the coding region of the *MaMsn2* gene was amplified and inserted into PgpdM-bar-*egfp* and PgpdM-Sur, respectively, then the recombinant plasmids were transformed into wild type strain and Δ*MaH1*, respectively. All primers used in this study are listed in Supplementary Table S1.

### 2.4. Conidial Development

Conidia suspension (10^7^ conidia/mL) was prepared with 0.05% Tween 80. Aliquots of 100 μL conidial suspension were spread onto plates evenly. Small pieces of agar media containing fungal colonies were cut at regular intervals. Conidial development was observed under microscope (Leica, Weztlar, Germany) and photographed. 

### 2.5. Conidial Yield and Stress Assay

To measure the conidial yield of the different strains, 1 mL of 1/4 SDAY or SYA medium was added to each well of the 24-cell plates. Two microliters of conidia suspension (10^6^ conidia/mL) of fungal strains was inoculated into each well, and the plates were incubated at 28 °C for 15 days. Conidia of each strain were collected with sterile 0.05% Tween 80 from three wells every 3 days from the 3rd day. The conidia quantity was determined by a hemocytometer. All experiments were performed in triplicate. 

The tolerance of conidia to heat shock and UV-B radiation was carried out according to a previous report [8]. 

### 2.6. Bioassay

The bioassay with 5th instar nymph of *Locusta migratoria* was conducted by a previous method [36]. Briefly, for topical inoculation, 10^7^ conidia/mL conidial suspensions of *M. acridum* were prepared with paraffin oil, and 5 µL was dripped onto the head–thorax junction of insects. The blank control was dripped with paraffin oil. Mortality of locusts was determined every 12 h until all locusts in each treatment died. Thirty locusts were used in each group with three groups for each treatment. The bioassay was performed in triplicate.

### 2.7. RNA Isolation and Real-Time qPCR (RT-qPCR)

Total RNA extraction was performed using Ultrapure RNA Kit (CWBIO, Beijing, China) according to the manufacturer’s instructions. RNAs were reverse transcribed into cDNAs using PrimeScript™ RT Master Mix (TAKARA, Dalian, China). Quantitative PCR analysis was performed with TB Green qPCR Master Mix (TAKARA, Dalian, China) with paired primers. The reference gene is glyceraldehyde 3 phosphate dehydrogenase (GAPDH). The transcription level of each gene was calculated according to the 2^−(ΔΔCt)^ method [37]. Transcriptions of conidiation-related genes *MedA* (XM_007809331.1), *Som1* (XM_007810626.1), *StuA* (XM_007811978.1) and *AbaA* (XM_007814389.1) were analyzed by RT-qPCR. These four genes have been reported to be involved in conidiation in other fungi and *M. acridum* by our group. The experiments included three replicates. 

### 2.8. Yeast One-Hybrid Assay (Y1H)

Y1H assay was conducted using Matchmaker^®^ One-Hybrid Library Construction & Screening Kit (Cat. No.PT3529-1, TAKARA, Dalian, China) according to the manufacturer’s instructions. Simply put, the *MaMsn2* promoter fragment was ligated to the pHis2.1 vector and then the recombinant vector pHis2.1-promoter was transformed into Y187 cells and screened on the SD-His-Trp with the appropriate 3-amino-1,2,4-triazole (3-AT) working concentration. The cDNA of *MaH1* was linked to the pGADT7 vector to form pGADT7–MaH1. pHis2.1-promoter and pGADT7–MaH1 vectors were co-transformed into Y187, and then Y187 was cultured under the 3-AT working concentration selected above on SD-His-Trp-Leu medium. p53HIS2 and pGAD-Rec-53 were positive controls, and pHis2.1 and pGADT7–MaH1 served as negative controls.

### 2.9. EMSA Assay

The sequence of the DNA binding domain of MaH1 was ligated to the pET-32a vector. After sequencing, the recombinant plasmid was introduced into *E. coli* transetta (DE3). The recombinant MaH1 protein (rMaH1) expression was induced by 0.5 mM IPTG at 18 °C. rMaH1 was purified by Ni^2+^ affinity chromatography (Cat. No. DP101-02, Transgen, Beijing, China) and preserved at −80 °C. EMSA was conducted using EMSA Probe Biotin Labeling Kit and Chemiluminescent EMSA Kit (Cat. No. GS008, GS009, Beyotime, Shanghai, China). Briefly speaking, the fragments of different *MaMsn2* promoter regions were biotin-labeled, respectively, and used as probes. The probes and the protein were incubated at 25 °C for 30 min. The reactants were separated by PAGE electrophoresis at 80 V for 90 min, and then proteins and DNAs were transferred to nylon membrane using a wet transfer unit (Bio-Rad, Hercules, CA, USA) at a constant voltage of 100 V for 50 min. The nylon membrane was then cross-linked by UV radiation. Finally, the Chemiluminescent EMSA Kit (GS009, Beyotime, Shanghai, China) was used to detect the binding of protein and probe. 

## 3. Results

### 3.1. Bioinformatic Analysis and Deletion of MaMsn2.

**Figure 1:**
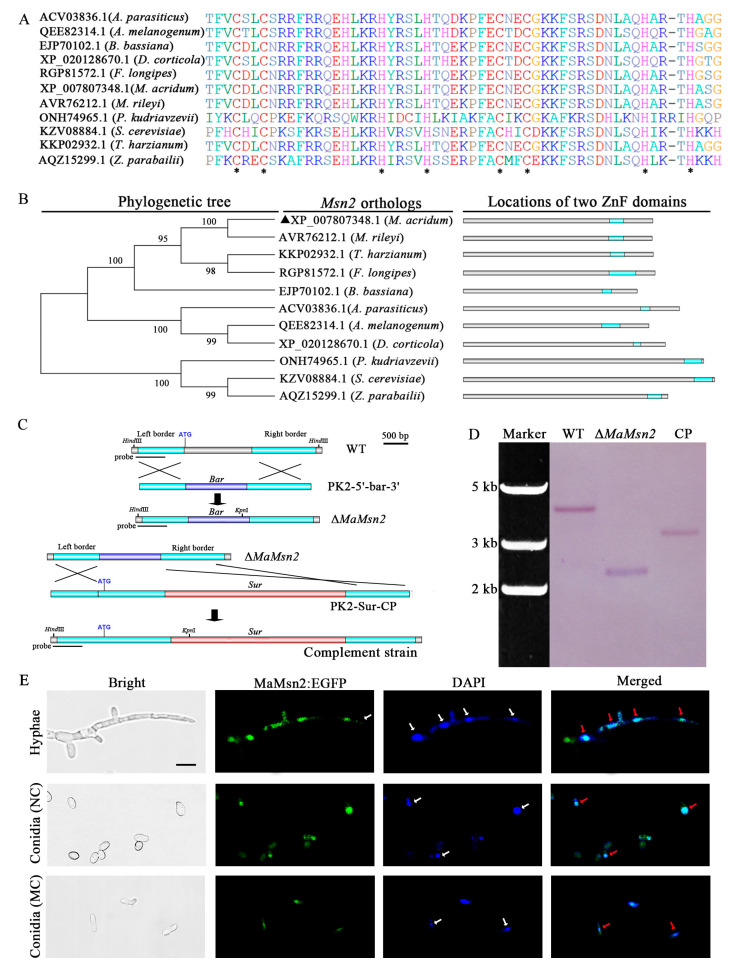
Sequence analysis and intracellular localization of MaMsn2. (**A**) Msn2 protein sequence alignment. The Msn2 homologous genes are from *Aspergillus parasiticus*, *Aureobasidium melanogenum*, *Beauveria bassiana* ARSEF 2860, *Diplodia corticola*, *Fusarium longipes*, *M. acridum* CQMa 102, *M. rileyi*, *Pichia kudriavzevii*, *Saccharomyces cerevisiae*, *Trichoderma harzianum* and *Zygosaccharomyces parabailii*. Asterisks indicate conservative C and H loci. (**B**) Main domains and phylogenetic relationships of Msn2. ▲: Msn2 in *M. acridum*. (**C**) The knockout (upper) and complement (lower) schematic diagram of *MaMsn2.* (**D**) The Southern blot verification of Δ*MaMsn2* and CP strains. Restriction enzymes *Hind*III and *Kpn*I were used to digest genomic DNAs. Probe location is shown in the diagram. (**E**) LSCM images of subcellular localization of MaMsn2:EGFP. Hyphae were collected from 1/4 SDAY grown for 18 h and conidia were collected on 1/4 SDAY and SYA grown for 48 h. NC: Conidia produced on 1/4 SDAY, MC: Conidia produced on SYA. White arrows: DAPI-stained nuclei, red arrows: merged nuclei. Scale bar indicates 5 μm.

The *Msn2* homologous gene in *M. acridum* (*MaMsn2,* Acc No. MZ556966) has a 1751 bp open reading frame (ORF) and is interrupted by an intron of 152 bp. It encodes a protein of 532 amino acids with a predicted pI of 4.77 and a molecular weight of 56.89 kDa (https://web.expasy.org/protparam/, accessed on 12 November 2019). Multiple sequence alignment showed that MaMsn2 has two C_2_H_2_ zinc finger structures in tandem at the C-terminal (Figure 1A,B) with a close relationship to that of *Metarhizium rileyi* (Figure 1B). In order to analyze the function of *MaMsn2*, we constructed a *MaMsn2* knockout mutant (Δ*MaMsn2*). The *MaMsn2* knockout and complement schematic diagrams are shown in Figure 1C. Southern blot confirmed the correct targeting in Δ*MaMsn2* and the insertion of the *MaMsn2* complement cassette in complemented strains (CP) (Figure 1D). 

In order to explore the localization of MaMsn2 in cells, we fused *MaMsn2* with *egfp* at the C-terminal and analyzed the localization of MaMsn2 by laser scanning confocal microscopy (LSCM). The results showed that the EGFP signal was overlapped with the DAPI nuclear staining in hyphae and conidia (produced from NC or MC pattern), indicating that MaMsn2 was located in the nucleus, likely to be a transcription factor (Figure 1E). 

### 3.2. MaMsn2 Affects Germination and Conidial Yield in M. acridum

**Figure 2:**
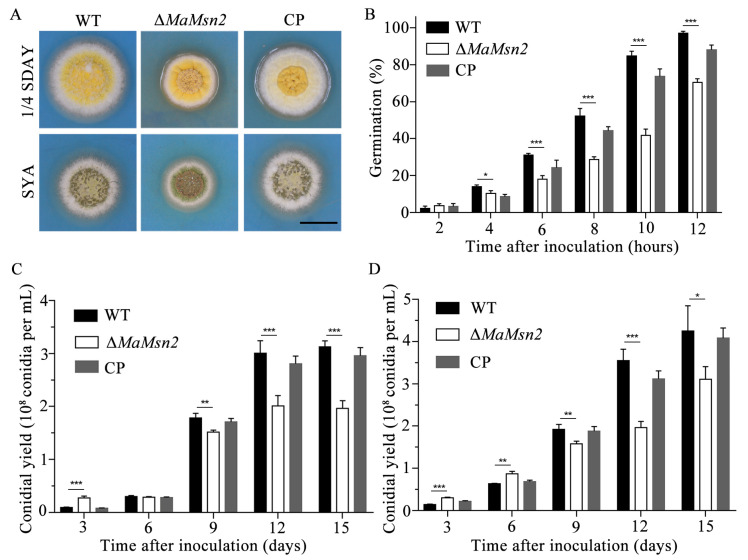
Growth and conidiation analysis. (**A**) The colonies of Δ*MaMsn2* grown on 1/4 SDAY and SYA media for 5 days. The scale bar indicates 1 cm. (**B**) Conidial germination rate of Δ*MaMsn2* for 2, 4, 6, 8, 10 and 12 h. (**C**,**D**) Conidial yields of Δ*MaMsn2* at 3, 6, 9, 12 and 15 days on 1/4 SDAY (**C**) and SYA (**D**). (*t*-test, *: *p* < 0.05, **: *p* < 0.01, ***: *p* < 0.001).

In order to explore the function of *MaMsn2* in the germination and conidiation of *M. acridum*, we measured the germination rate and the conidial yields of Δ*MaMsn2* on 1/4 SDAY (nutrient-rich medium) and SYA (nutrient-limited medium). Δ*MaMsn2* had a significantly smaller colony and a drastically wrinkled colonial surface compared to the wild type on both 1/4 SDAY and SYA plates (Figure 2A). The germination rate of Δ*MaMsn2* was significantly lower than that of the wild type since the 4th hour of culture. At the 12th hour, the germination rate of Δ*MaMsn2* was 70% while the WT strain had reached almost 100% (Figure 2B). The conidial yield of Δ*MaMsn2* was significantly lower than that of WT and CP strains on 1/4 SDAY (Figure 2C) and SYA media (Figure 2D). Interestingly, at the early stage of conidiation, e.g., on the 3rd day on 1/4 SDAY and 3rd day and 6th day on SYA, the conidial yield of Δ*MaMsn2* was significantly higher than that of the WT, but was subsequently exceeded by the WT during the following growth period (Figure 2C,D).

### 3.3. MaMsn2 Affects Multiple Stress Responses and Virulence in M. acridum

**Figure 3:**
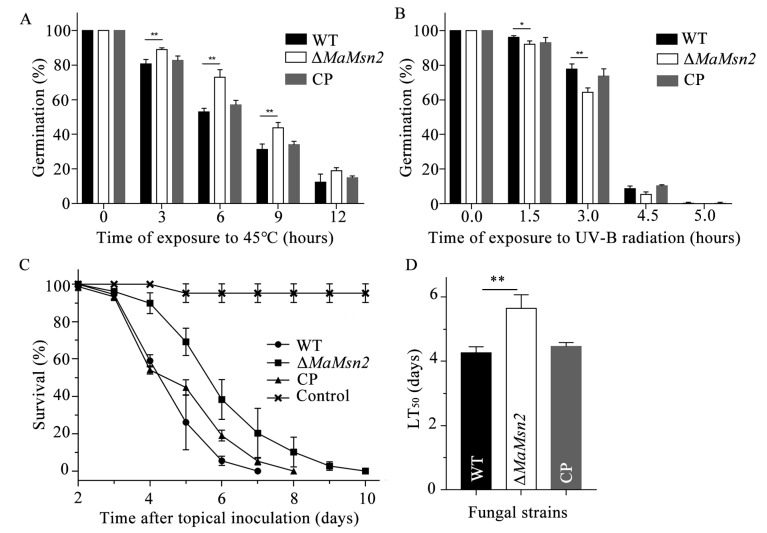
Stress tolerance and insect bioassays. (**A**) Conidial germination rate of Δ*MaMsn2* after heat shock treatment at 45 °C for 0, 3, 6, 9 and 12 h. (**B**) Conidial germination rate of Δ*MaMsn2* after UV-B treatment at 1350 mW/m^2^ for 0, 1.5, 3.0, 4.5 and 6.0 h. (**C**) Survival of the locusts following topical inoculation with 5 μL aqueous conidial suspensions of 1×10^7^ conidia/mL of WT, Δ*MaMsn2* and CP strains. Control insects were treated with 5 μL paraffin oil. (**D**) LT_50_ of different strains in topical inoculation. (*t*-test, *: *p* < 0.05, **: *p* < 0.01).

In order to explore the roles of *Msn2* in fungal stress tolerance, the germination rate of conidia was determined after heat shock and UV-B stress treatment. The results showed that Δ*MaMsn2* had a significantly increased germination rate after 3, 6, 9 and 12 h of treatment at 45 °C (Figure 3A). However, Δ*MaMsn2* had a significantly decreased germination rate after exposure to UV-B irradiation (Figure 3B), suggesting a positive role of *MaMsn2* in UV-B stress tolerance and a negative role in heat shock stress tolerance. 

In order to explore the effect of *MaMsn2* on the virulence of *M. acridum*, a bioassay was conducted using 5th instar nymphs of *Locusta* by topical inoculation. The results showed that the virulence of Δ*MaMsn2* was significantly lower than that of the WT (Figure 3C), and the half-lethal time (LT_50_) of Δ*MaMsn2* was 1.4 days longer than that of the WT strain (Figure 3D). 

### 3.4. MaMsn2 Regulates Conidiation Pattern Shift of M. acridum

**Figure 4:**
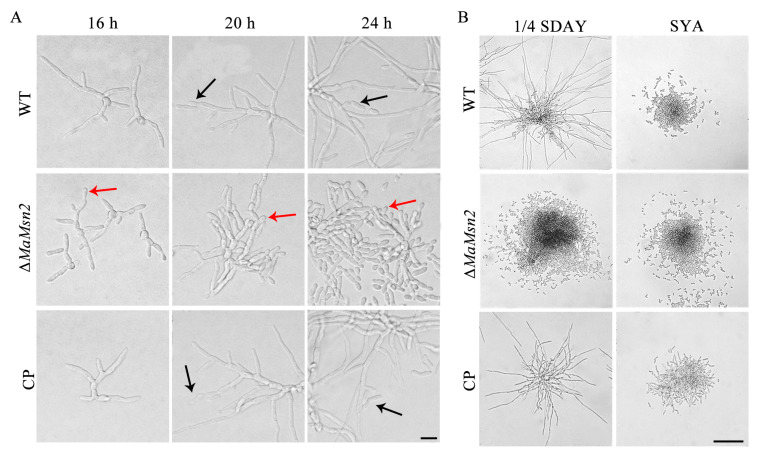
The conidiation phenotype of Δ*MaMsn2*. (**A**) The conidiation of Δ*MaMsn2* on 1/4 SDAY medium at 16, 20 and 24 h. Scale bars indicate 5 μm. Black arrow: normal conidiation; Red arrow: microcycle conidiation. (**B**) The conidiation phenotype of Δ*MaMsn2* on 1/4 SDAY and SYA media for 32 h. Scale bars indicate 100 μm.

*M. acridum* conducts normal conidiation on 1/4 SDAY and microcycle conidiation on the SYA medium [8]. In order to analyze the effects of *MaMsn2* on the conidiation pattern of *M. acridum*, conidiation phenotypes of Δ*MaMsn2* were observed on 1/4 SDAY and SYA, respectively. On the 1/4 SDAY medium, Δ*MaMsn2* produced conidia by microcycle conidiation (Figure 4A), with a drastic suppression of the mycelial growth and extension compared to that of WT and CP strains (Figure 4B). On the SYA medium, Δ*MaMsn2* conducted microcycle conidiation with no mycelia as the WT strain (Figure 4B). These results indicated that *MaMsn2* was essential for NC and regulated the conidiation pattern shift in *M. acridum* on 1/4 SDAY.

### 3.5. MaMsn2 Is Regulated by MaH1

**Figure 5:**
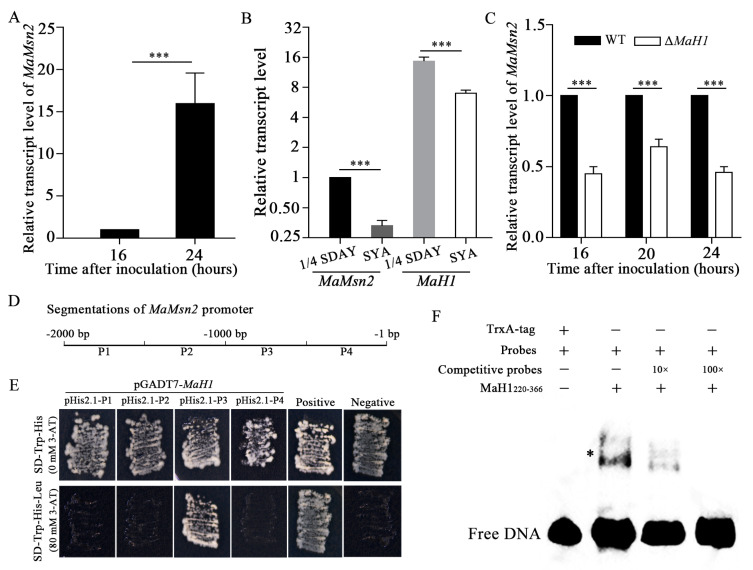
Interaction analysis between MaH1 and *MaMsn2*. (**A**) Transcription level of *MaMsn2* in WT on 1/4 SDAY at 16 h and 24 h. The *MaMsn2* transcript level at 16 h was set to 1. (*t*-test, ***: *p* < 0.001). (**B**) Transcription levels of *MaH1* and *MaMsn2* in WT on 1/4 SDAY and SYA at 24 h. The *MaMsn2* transcript level on 1/4 SDAY was set to 1. (*t*-test, ***: *p* < 0.001) (**C**) Transcription levels of *MaMsn2* in WT and Δ*MaH1* on 1/4 SDAY at 16 h, 20 h and 24 h. (*t*-test, ***: *p* < 0.001). (**D**) Different regions of *MaMsn2* promoters for binding analysis with MaH1 by Y1H. (**E**) Y1H assay between MaH1 and different *MaMsn2* promoter regions. (**F**) EMSA assay with purified trxA-MaH1^220−366^ and probe labeled by biotin. The competitive probe was unlabeled and 10 to 100 fold excess compared to the labeled probe. +: probe or protein added, –: probe or protein not added. Asterisk indicates the position of shifted band.

Our previous study showed that *MaH1* was involved in the conidiation pattern shift in *M. acridum*. Similar to Δ*MaMsn2*, Δ*MaH1* also exhibited a microcycle conidiation pattern on nutrient-rich medium 1/4 SDAY [22]. Therefore, we would like to know whether these two genes directly regulate conidiation and whether they have any interaction. First of all, we analyzed the transcription level of *MaMsn2* in the WT on 1/4 SDAY at 16 h and 24 h after inoculation, which are the hyphal stage and the beginning of normal conidiation, respectively (Figure 4A). The result showed that the transcription level of *MaMsn2* increased by 16 times at 24 h compared to 16 h, suggesting that Msn2 was involved in conidiation (Figure 5A). To compare the transcription levels of *MaMsn2* and *MaH1* on 1/4 SDAY and SYA, we determined their transcriptions at 24 h after inoculation. At this time point, *M. acridum* conducts normal conidiation on 1/4 SDAY and microcycle conidiation on SYA. The results showed that the transcription levels of *MaMsn2* and *MaH1* were significantly higher on 1/4 SDAY than that on SYA (Figure 5B). More importantly, quantitative PCR analysis showed that the transcription level of *MaMsn2* was significantly downregulated in Δ*MaH1* (Figure 5C). Therefore, MaH1 might regulate conidiation via *MaMsn2*. In order to confirm this speculation, we performed Y1H and electrophoretic mobility shift assay (EMSA) analysis. Four regions (P1, P2, P3, P4) in the *MaMsn2* promoter were assessed, respectively, for a possible interaction with MaH1 (Figure 5D). 

**Figure 6:**
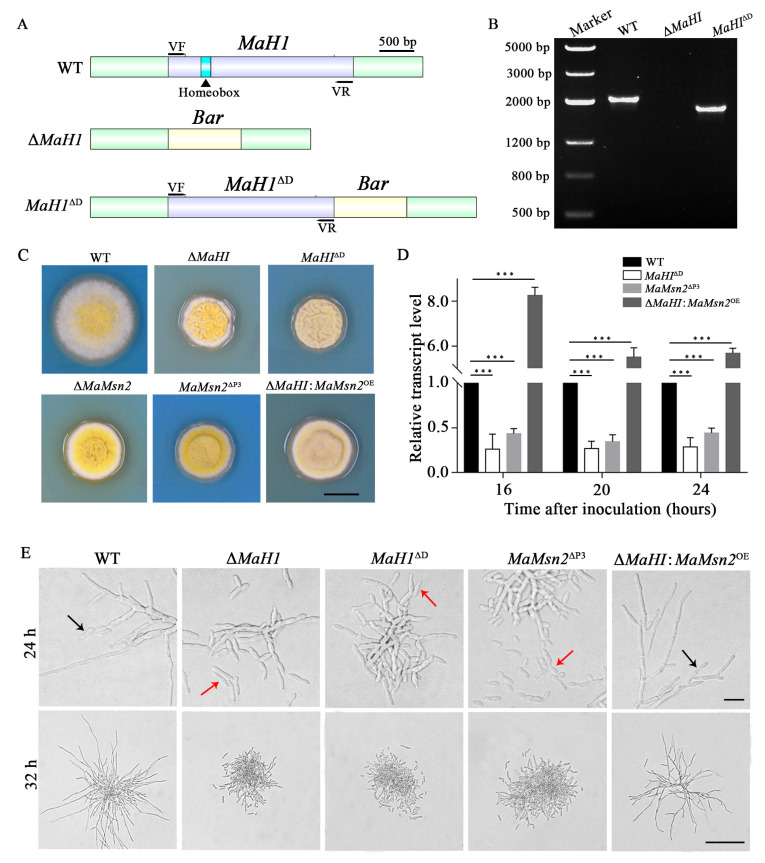
Complementation of Δ*MaH1* by *MaMsn2*. (**A**) Schematic diagram of deletion of the *MaH1* DNA binding domain. (**B**) PCR verification of DNA binding domain knockout strain *MaH1*^ΔD^. Results show a fragment 135 bp smaller in *MaH1*^ΔD^ than that of the wild type. (**C**) Colony morphology of Δ*MaH1*, DNA binding domain knockout strain of *MaH1* (*MaH1*^ΔD^), Δ*MaMsn2*, promoter P3 region knockout strain of *MaMsn2* (*MaMsn2*^ΔP3^) and Δ*MaH1* strain overexpressing *MaMsn2* (Δ*MaH1*:*MaMsn2*^OE^). Scale bars indicate 0.5 cm. (**D**) The transcript level of *MaMsn2* on 1/4 SDAY medium at 16, 20 and 24 h in *MaH1*^ΔD^, *MaMsn2*^ΔP3^ and Δ*MaH1*:*MaMsn2*^OE^ strains (*t*-test, ***: *p* < 0.001). (**E**) The conidiation and hyphal development of different strains. Black arrow: normal conidiation; Red arrow: microcycle conidiation. The scale bars indicate 5 μm at 24 h and 100 μm at 32 h.

The Y1H result showed that the MaH1 protein directly interacted with the P3 region (−1000 bp to −500 bp), but did not bind to the P1, P2 and P4 regions of the *MaMsn2* promoter (Figure 5E). The EMSA result showed that MaH1^220–366^ containing the DNA binding motif homeobox could bind to a 40 bp fragment of −920 to −880 bp of the *MaMsn2* promoter (Figure 5F). These results indicated that MaH1 could directly bind to the *MaMsn2* promoter cis-element in *M. acridum*. In order to further verify the interaction between the binding domain of MaH1 and *MaMsn2*, we constructed a homeobox domain deletion mutant (*MaH1*^ΔD^) (Figure 6A), an engineered strain overexpressing MaMsn2 in Δ*MaH1* (Δ*MaH1*:*MaMsn2*^OE^) and a promoter P3 region deletion mutant (*MaMsn2*^ΔP3^). PCR analysis with VF/VR primer pairs confirmed that the homeobox domain region was deleted (Figure 6B). Similar to Δ*MaMsn2,* the Δ*MaH1*, *MaH1*^ΔD^ and *MaMsn2*^ΔP3^ colonies all had significantly smaller size with more wrinkles compared to the WT. Meanwhile, the colony size of Δ*MaH1*:*MaMsn2*^OE^ was between Δ*MaH1* and the WT, and the wrinkles disappeared in Δ*MaH1*:*MaMsn2*^OE^, suggesting a complementation of the defect in Δ*MaH1* by overexpressing *MaMsn2* (Figure 6C).

RT-qPCR analysis showed that the transcription of *MaMsn2* was significantly downregulated in *MaH1*^ΔD^ and *MaMsn2*^ΔP3^, and significantly upregulated in the Δ*MaH1*:*MaMsn2*^OE^ strain compared to the WT (Figure 6D). Consistent with Δ*MaH1* and Δ*MaMsn2*, *MaH1*^ΔD^ and *MaMsn2*^ΔP3^ showed microcycle conidiation on 1/4 SDAY, while the conidiation of Δ*MaH1*:*MaMsn2*^OE^ shifted to normal conidiation, indicating that *MaMsn2* compensated for the conidiation defect of Δ*MaH1* (Figure 6E). These results suggested that the homeobox domain in MaH1 was essential for activating the transcription of *MaMsn2*. 

### 3.6. ΔMaMsn2 and MaH1^ΔD^ Had Similar Phenotypes 

**Figure 7:**
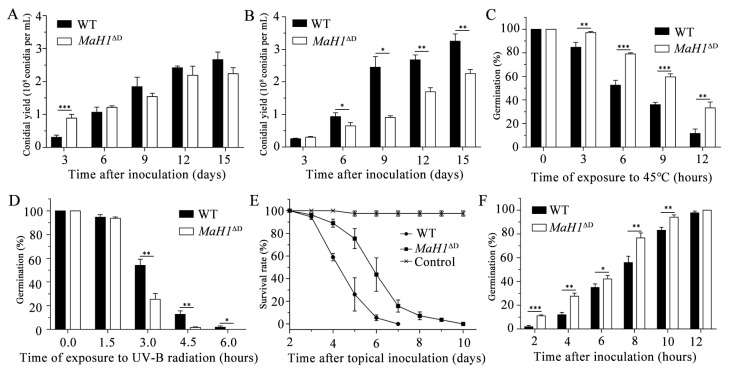
Phenotypes of *MaH1*^ΔD^. (**A**,**B**) Conidial yields of *MaH1*^ΔD^ at 3, 6, 9, 12 and 15 days on 1/4 SDAY (**A**) and SYA (**B**). (**C**) Conidial germination rate of *MaH1*^ΔD^ after heat shock treatment at 45 °C for 0, 3, 6, 9 and 12 h. (**D**) Conidial germination rate of *MaH1*^ΔD^ after UV-B treatment at 1350 mW/m^2^ for 0, 1.5, 3.0, 4.5 and 6.0 h. (**E**) Survival of the locusts following topical inoculation with 5 μL aqueous conidial suspensions of 1 × 10^7^ conidia/mL of WT and *MaH1*^ΔD^ strains. Control insects were treated with 5 μL paraffin oil. (**F**) Conidial germination rate of *MaH1*^ΔD^ for 2, 4, 6, 8, 10 and 12 h (*t*-test, *: *p* < 0.05, **: *p* < 0.01, ***: *p* < 0.001).

Interaction assay and RT-qPCR analysis demonstrated that MaH1 directly regulated the transcription of *MaMsn2*. We then measured the conidial yield, tolerance to heat shock and UV-B radiation stresses, virulence and germination rate of *MaH1*^ΔD^ and compared those results with Δ*MaMsn2*. The results showed that *MaH1*^ΔD^ had a similar trend to that of Δ*MaMsn2* in determined phenotypes, such as lower conidial yields (Figure 7A,B), stronger resistance to heat shock (Figure 7C), weaker anti-UV-B irradiation ability (Figure 7D) and decreased virulence with topical inoculation (Figure 7E). However, the germination of *MaH1*^ΔD^ was earlier than that of the WT (Figure 7F), which was opposite to Δ*MaMsn2*. These similar phenotypes between Δ*MaMsn2* and *MaH1*^ΔD^ also indicated the direct regulation of MaH1 on *MaMsn2*.

### 3.7. MaMsn2 Affected the Expression of AbaA and StuA 

**Figure 8:**
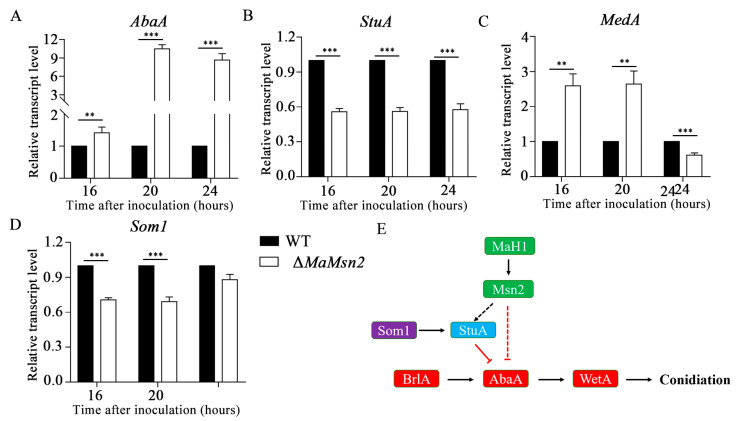
*MaMsn2* regulates *AbaA* and *StuA*. (**A**–**D**) The relative expressions of conidiation-related genes in Δ*MaMsn2* (*t*-test, ** : *p* < 0.01, *** : *p* < 0.001). (**E**) Genetic model for *MaH1*–*MaMsn2* in conidiation pathway.

In order to explore the effects of *MaMsn2* on conidiation, we measured the time-course transcription level of some conidiation-related genes in Δ*MaMsn2*. The results showed that *AbaA* significantly increased in Δ*MaMsn2* (Figure 8A), while *StuA* was down-regulated in Δ*MaMsn2* (Figure 8B). The transcriptions of other conidiation-related genes, such as *MedA* and *Som1*, did not show consistent changes in Δ*MaMsn2* compared to the WT in the conidiation period (Figure 8C,D). These results indicated that *MaMsn2* negatively regulated the transcription of *AbaA* and positively regulated *StuA*. The prediction of conserved binding sites against the Jaspar 2020 database [38] showed that *Msn2* recognition sites were present on *AbaA* and *StuA* promoters (data not shown) but not on *MedA* or *Som1* promoters. Combining all the above results*,* we can infer that the *MaH1*–*MaMsn2* pathway affected the central conidiation pathway *BrlA*–*AbaA*–*WetA* by *AbaA* and *StuA* (Figure 8E).

## 4. Discussion

*Msn2* and *Msn4* genes widely exist in fungi, and were first reported in yeast as homologous genes. They have a C_2_H_2_-type zinc finger structure and participate in different stress responses, such as glucose starvation, heat shock and osmotic and oxidative stress. The transcriptions of stress-related genes CTTI, DDR2 and HSP12 can be regulated through stress response elements by *Msn2/4* [39]. However, in filamentous fungi, only Msn2 gene was present while Msn4 has not been found [40]. Consistent with *Msn2* homologous genes in other fungi [23,24], MaMsn2 is mainly located in the nucleus in *M. acridum*. 

*Msn2* plays an important role in stress response through multiple pathways. In yeast cells, the Ras–cAMP–PKA pathway can regulate the oxidative stress response through *Msn2/4* [41], and the high osmolarity glucose (HOG) pathway can also regulate the activity of *Msn2/4* [25]. The deletion of the *BbMsn2*, *MrMsn2* or *MoMsn2* gene reduced the stress resistance to varying degrees in *B. bassiana*, *M. robertsii* and *M. oryzae* [31,33]. Our study found that the anti-UV-B ability of Δ*MaMsn2* slightly decreased, which is consistent with other fungal *Msn2*. However, different from previous reports, the resistance of Δ*MaMsn2* to heat shock increased significantly, indicating that *Msn2* functions differently in regulating the response to heat stress in different fungal strains. Our data showed that MaMsn2 was also a virulence factor. Loss of *MaMsn2* resulted in decreased virulence. Similar results have been reported in *B. bassiana* and *M. robertsii* [31], *M. oryzae* [33] and *V. dahliae* [34].

*Msn2* is regulated in the process of carbon and nitrogen utilization. TOR1, which is involved in nitrogen source utilization, upregulates the expression level of phosphatase PP2A, which then promotes the nucleus localization of Msn2 through phosphorylation [24,28]. Under low glucose conditions, Msn2 is phosphorylated by the activated protein kinase Snf1 and located in the cytoplasm [23,27]. Microcycle conidiation, as a typical characteristic of filamentous fungi, can be induced by the change in the nutrient composition. On the nutrient-rich 1/4 SDAY medium, the wild type *M. acridum* firstly grows radial polar hyphae, and then produces conidia on top or both sides of the hyphae. However, Δ*MaH1* and Δ*MaMsn2* perform microcycle conidiation without hyphal growth on 1/4 SDAY (Figure 6E). Homeobox proteins are widely involved in the hyphal development and conidiation process of filamentous fungi [19,20,21,42,43,44]. In *M. oryzae*, homeobox protein Htf1 may interact with *Acr1* to regulate the conidiation process [45]. *Msn2* also regulates the hyphal growth and conidial yield in filamentous fungi such as *M. oryzae*, *V. dahliae* and *B. bassiana* [31,33,34]. Similar to other fungi, our data indicate that *MaMsn2* can participate in the nutritional perception process and regulate the process of hyphal development and conidiation in *M. acridum*.

Our data show that Δ*MaH1* and Δ*MaMsn2* have a similar conidial phenotype on 1/4 SDAY, suggesting that these two genes are probably in the same regulatory network in the conidial process. The results of qPCR, Y1H and EMSA proved that *MaMsn2* is directly regulated by MaH1 in conidiation (Figure 5). Previous studies showed that *MaH1* affects conidiation in *M. acridum,* but does not affect resistances to heat shock, UV-B radiation and virulence [22]. On the contrary, *MaMsn2* regulates stress resistances and virulence, indicating that *MaMsn2* was not regulated by *MaH1* in these two processes. When the DNA binding domain of *MaH1* was deleted (*MaH1*^ΔD^), the shortened MaH1 could not bind to the promoter region of *MaMsn2.* Meanwhile, when the promoter region −1000 to −500 bp of *MaMsn2* was deleted (*MaMsn2*^ΔP3^), *MaMsn2* was significantly downregulated. *MaH1*^ΔD^ exhibited similar phenotypes to Δ*MaMsn2* and *MaMsn2*^ΔP3^ (Figure 7).

*AbaA* is a key gene in asexual conidiation in filamentous fungi. The disruption of *AbaA* leads to an abnormal conidiophore with an “abacus” phenotype [46,47,48]. *StuA*, an APSES (APSES: Asm1p, Phd1p, Sok2p, Efg1p and StuAp) transcription factor, is necessary for the spatial expression of *BrlA* and *AbaA* [49]. The deletion of *StuA* leads to a very short conidiophore, a lack of metulae and phialides and the formation of conidia directly from vesicles, showing a “stunted” phenotype. The high expression level of *StuA* inhibits the expressions of *BrlA* and *AbaA* [50]. Here, the disruption of *MaMsn2* leads to limited hyphal growth and a promoted conidiation process, indicating that *MaMsn2* plays a role in maintaining vegetative growth in *M. acridum*. Based on the qPCR result, *MaMsn2* has a strong negative effect on the expression of *AbaA*, while it has a positive regulatory role on *StuA*. In addition, conserved binding sites of Msn2 were found on the promoter region of *AbaA* and *StuA* genes [38]. Therefore, we infer that *MaMsn2* can either directly regulate the expressions of *AbaA* and *StuA* or *MaMsn2* positively regulated the expression of *StuA*. (Figure 8E). These results suggest that *MaMsn2* might participate in the conidiation process of *M. acridum* by regulating the transcription of *AbaA* directly or through *StuA*. Our analyses expanded the regulatory network of the central conidiation pathway from *BrlA*–*AbaA*–*WetA* to *MaH1*–*MaMsn2*–*AbaA*/*StuA*.

Microcycle conidiation is more applicable in mass production and field application compared with normal conidiation [8]. In this study, we found that *MaMsn2* can regulate the conidiation pattern shift in *M. acridum*, and was directly controlled by another conidiation-related protein MaH1. Our work reports the regulatory mechanism of MC for the first time, which enriches the knowledge of microcycle conidiation and dimorphism in filamentous fungi.

## Data Availability

The data presented in this study are available in this article and its Supplementary Materials.

## Supplementary Materials

The following are available online at www.mdpi.com/article/10.3390/jof7100840/s1, Table S1: Paired primers used in this paper.
