# Supplementary material for: Transcription Factor MaMsn2 Regulates Conidiation Pattern Shift under the Control of MaH1 through Homeobox Domain in Metarhizium acridum
Source: J Fungi (Basel). 2021 Oct 7;7(10):840. doi: 10.3390/jof7100840 (PMC8541488; doi:10.3390/jof7100840)
Supplement: Supplementary file 1 [file jof-07-00840-s001.zip › jof-1380977-supplementary.pdf]

Table S1. Paired primers used in this paper.

Primer	Paired sequences (5′–3′)	Purpose
Msn2-LF	gacggccagtgccaagcttCGAGTGAGACAAGCGTGGAG	*MaMsn2* disruption mutant construction
Msn2-LR	cggatccctcgagtctagaCATTATGGGAAGATGGATTAGC	
Msn2-RF	gctggccgcccatgggatatcGTCACCCAGCCCTCCCTCAA	
Msn2-RR	atgacatgattacgaattcCCCAAGCATCCTCGGCAAAT	
CP-Msn2-LF	gacggccagtgccaagctCGAGTGAGACAAGCGTGGAG	Complementation for Δ*MaMsn2*
CP-Msn2-LR	ccatggcggccgggagcaTTCAAAACGCTTTCGCTTCT	
CP-Msn2-RF	cgttggcacgtcgacgatGTCACCCAGCCCTCCCTCAA	
CP-Msn2-RR	atgacatgattacgaattCCCAAGCATCCTCGGCAAAT	
Msn2-ProbeF	CACCACTCACCATCCTCCAT	Probe for Southern blot
Msn3-ProbeR	TCCCAAAGTTGCCTCCAC	
Msn2pLF	gacggccagtgccaagctAGGCCAGAGAGTGCGCTG	Knockout for *MaMsn2*$^{\Delta P3}$
Msn2pLR	cggatccctcgagtctagGAATCTACAACACGATACG	
Msn2pLF	gctggccgcccatgggatatcGTCACCCAGCCCTCCCTCAA	
Msn2pLR	atgacatgattacgaattcCCCAAGCATCCTCGGCAAAT	
HDF1	gacggccagtgccaagctCTCATCTCCATCTCTCACC	Knockout for *MaH1*$^{\Delta D}$
HDR1	AACAACTTGATTTTGGCTCGTCTTTGTCTTTTCTGGTTCTTC	
HDF2	GAAGAACCAGAAAAGACAAAGACGAGCCAAAATCAAGTTG TT	
HDR2	cggatccctcgagtctagTCACGTAGGATGCGCAAC	
HDRF	gctggccgcccatgggatTTCGTTCCCAGTCTAGTGG	
HDRR	atgacatgattacgaattCACAAGTATGAACCAAGTCC	
Msn2-oesur-F	tacacacacgcaaatctagaATGGACTCAAACACGATGCC	Overexpressing *MaMsn2* in Δ*MaH1*
Msn2-oesur-R	ctcaccatactagtctcgagTTCAAAACGCTTTCGCTTCTTC	
qF-MedA	GTCTTGTCAGCCAATGTCGG	RT-qPCR for detection
qR-MedA	CCAATAGTTGCGGAGCGTCTA	

qF-StuA	CCAGGTAATCCAGGCGTAGG	conidiation-related genes
qR-StuA	TGAGAAGCAGAAGCACCATACA	
qF-Sho1	TCGGTGTTGTAGTGGTGGTC	
qR-Sho1	GGTGTTGAAGAAGGCGTTGAG	
qF-Som1	AAGGACGGAAAGAGTGGCAA	
qR-Som1	TGAGTGTGAGCGACATATTGGT	
qF-abaA	AAGAAGAGCAGGAGTCATTCAAGT	
qR-abaA	GAGCATCGTAGGCAACATCATC	
qF-MaH1	TTGGCGACTTCACAGAGGAT	
qR-MaH1	GACACAGGAGCAGACACAGA	
qF-MaMsn2	GGGTCATCCGTTGTTTCCTTG	
qR-MaMsn2	TTATCCGCAGCAGCAGTGTT	
